# Urinary retention induced by psychotropics: A case report

**DOI:** 10.1192/j.eurpsy.2024.1443

**Published:** 2024-08-27

**Authors:** E. Smaoui, D. Mnif, N. Reguaieg, F. Guermazi, S. Sakka, I. Baati, J. Masmoudi

**Affiliations:** ^1^Psychiatry, CHU Hédi Chaker; ^2^Neurology, CHU Habib Bourguiba, Sfax, Tunisia

## Abstract

**Introduction:**

Neurological bladder is considered a functional disability that has a significant impact on the quality of life and psychological state of patients. Psychotropic drugs, in turn, can worsen the urinary dysfunction caused by this disease.

**Objectives:**

Our objective is to illustrate, through the case of a patient suffering from a neurological bladder decompensated by the treatment of a characterized depressive episode, the link between these two pathologies.

**Methods:**

We report the case of Ms. M.W., aged 51, with a history of high blood pressure stabilized under nebivolol and a neurological bladder diagnosed 10 years ago with episodic pollakiuria, admitted to the psychiatric department for repeated suicide attempts. She had never used psychoactive substances and had no family psychiatric history. The patient presented depressive symptoms evolving for 5 months. The diagnosis of a characterized depressive episode with melancholic features was made and the patient was treated with sertraline. From the first intake of the drug, the patient presented acute urinary retention (UR) requiring the placement of a permanent bladder catheter. The urinary symptoms improved upon stopping the treatment. Sertraline was changed to olanzapine and escitalopram. The patient stopped the treatment after one month because of the worsening of urinary symptoms requiring the installation of a suprapubic catheter. The urinary problem, together with the cessation of treatment, were responsible for a worsening of psychiatric symptoms leading to multiple suicide attempts. Given the advanced stage of the neurological bladder demonstrated by the urodynamic tests, our patient was treated with paroxetine, quetiapine and oxazepam along with psychotherapeutic education. The evolution was characterized by improvement in psychiatric symptoms and the urinary symptoms were stable.

**Results:**

The lack of improvement after treatment discontinuation could be explained by an underlying neurological bladder manifesting with pollakiuria. The current literature on UR induced by psychotropic treatments is quite rare limited in case reports. This effect occurs especially when selective serotonin reuptake inhibitors (SSRIs) are prescribed in combination with other antipsychotics. Unlike first generation antipsychotics, atypical antipsychotics have muscarinic receptor antagonist properties which can induce UR. Among atypical antipsychotics, olanzapine has been shown to have the greatest antimuscarinic effects. Regarding SSRIs, they are associated with a lower risk of UR than other antidepressants and sertraline had the highest risk of UR.

**Conclusions:**

SSRIs can induce UR particularly in combination with atypical antipsychotics. Coordination of care across multiple specialties and understanding the side effects of psychotropic medications can enable faster diagnoses and adequate management.

**Disclosure of Interest:**

None Declared

